# Preparation and thermal cross-linking mechanism of co-polyester fiber with flame retardancy and anti-dripping by *in situ* polymerization†

**DOI:** 10.1039/d1ra07410e

**Published:** 2021-12-20

**Authors:** Keyu Zhu, Zhenlin Jiang, Xiaotong Xu, Yun Zhang, Min Zhu, Jianghua Wang, Alex Ren

**Affiliations:** College of Chemistry and Chemical Engineering, Research Center for Advanced Mirco- and Nano-Fabrication Materials, Shanghai University of Engineering Sciences Shanghai 201620 PR China jiangzhenlin@sues.edu.cn; Science and Technology on Advanced Ceramic Fibers and Composites Laboratory, National University of Defense Technology Changsha 410073 PR China; Jiangsu Guowanggaoke Fiber Co., Ltd Suzhou 215228 PR China; Shanghai Rongteng Packing Service Co., Ltd Shanghai 201620 PR China

## Abstract

Extensive research has been conducted on polyester flame retardants and anti-droplet modifications in recent years. The conventional methods used to improve the effectiveness of the anti-droplet modifications usually involve improving the melt fluidity and the combustion char formation through reactive cross-linking. However, these methods, while reducing the droplets, may produce more smoke. This study proposes a combustion cross-linking method which avoids the droplet and flame retardancy synergistic modification problem. Based on the flame retardancy of polyester, anti-droplet properties were realized using a collaborative cross – linking structure formed by a phosphorus – containing flame – retardant group and acid silicon solvent to achieve a flame retardant and anti-droplets result. The results show that the phosphorus–silicon copolyester presents an enhancement effect for flame retardancy, confirmed by obvious reductions in the peak value of heat release rate (78.4%) and total heat release (44.2%). Meanwhile, the total smoke release and smoke product rate of phosphorus–silicon copolyester are decreased by 45.1% and 41.5%, respectively. And the phosphorus–silicon copolyester has a high LOI value of 34.8 ± 0.1% and UL-94 is V-0 rating with superior anti-dripping performance. Flame retardancy index (FRI) of the copolyesters containing phosphorus–silica are up to 4.3093 (good flame retardancy). Nonisothermal differential scanning calorimetry (DSC) was performed for qualitative analysis of network formation by the aid of Cure Index (CI) dimensionless criterion. It was observed that the acidic silica led to Excellent cure situation. The TG-DSC, XPS, and FTIR results validate the thermal cross-linking ability of the copolymer due to the synergistic cross-linking effect between the self-cross-linking characteristic of the catalysed acidic silica sol containing the phosphorus flame retardant. The SEM-EDX and Raman results further verify the effectiveness of the condensed-phase flame-retardant mechanism. Phosphorus–silicon copolyester has good spinnability, flame retardancy and anti-droplets properties. Which provides a simple method for preparing polyester by using this combustion synergistic crosslinking effect to achieve flame retardant and anti-dripping modification of copolymers.

## Abstract 2

Scheme of proposed thermal cross-linking mechanism.

## Graphical abstract

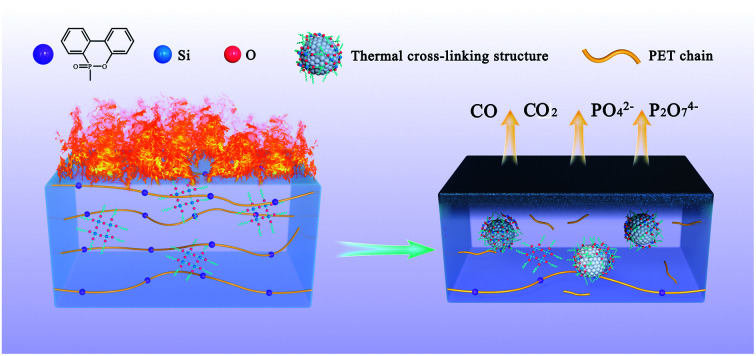

## Introduction

The burning of textiles is the most important factor leading to casualties in indoor fires, and about 50% of indoor fires are also caused by textiles. Extensive research has been conducted to focus on the flame retardancy of textile fabrics. Polyester has become the core raw material used in textile fabrics in recent years, and its flame retardancy and anti-dripping properties have remained key issues which should be resolved.^1–4^ The polyesters are inherently flammable and exhibit severe dripping behaviour during combustion, making them unsuitable for household textile decoration.^5–7^ Various approaches have been proposed in previous studies, including the addition of flame retardants into polyesters by blending, and incorporating flame-retardant monomers into the PET chains *via* polymerisation, and finishing the fabrics/textile in a solution containing flame retardants to improve the properties of retardancy.^8–10^ However, few solutions have been developed for these problems, owing to the large number of additives required, as well as the poor durability and compatibility of the materials. These issues have prompted the introduction of the phosphorus-containing monomers into the polyester backbone.^11–13^ The flame retardancy mechanism of these phosphorus-containing copolyesters is based on the removal of heat from the burning process, which results in an increase of the melt-dripping.^14,15^ Additionally, the conflict between the flammability and the melt-dripping characteristics presents a major limitation in the practical application of the flame-retardant polyester.

The severe melt-drop phenomenon of polyester is attributed to the combustion heat being greater than the melting enthalpy.^16^ Therefore, the blending and copolymerization are often selected for flame retardant fusion droplet modification. It has been reported that the copolymerisation of polyester with polytetrafluoroethylene, silicate, or magnesium hydrate can increase the residual carbon content and decrease the melt fluidity to improve the resistance of the droplets.^7,17^ Pyrolysis and fire behavior of phosphorus polyester (PET-P-DOPO) were studied by Pospiech *et al.*^18,19^ They modified PBT with a phosphorus polyester (PET-P-DOPO) as a halogen-free flame retardant. The effects of three different mechanisms (flame retardancy, carbonization and protection of expanded carbon) on the flame retardancy of polyethylene terephthalate were investigated. The result show that PET-P-DOPO showed excellent flame retardancy, in particular due to the additional prolongation of the time to ignition and increase in char yield. But they did not make a detailed analysis of meltdroplet. Yang *et al.*^20,21^ used carbon microspheres coated with magnesium hydroxide (Mg(OH)_2_@CMSs) to improve the fire performance of polyester, and Mg(OH)_2_@CMSs by promoting cross-linking of pyrolysis products and improving char layer continuity. Although the effect of anti-droplet can be achieved, the flame retardancy is very low. However, a large number of additives are required to achieve a good anti-melting effect. This leads to poor fluidity and affects the processing, which increases the difficulty of the application of the modified polyester in the melt-spinning process.^22,23^ Jiang *et al.*^24^ used polysiloxane and phytic acid as raw materials for flame retardant and anti-drop modification of polyester fabric. The results show that the polyester fabric has good flame retardancy and durability has been greatly improved. Wang *et al.*^25–28^ introduced unsaturated conjugated groups into the polyester chain segments (*e.g.* azobenyl and phenylethyl groups) to solve the melt-drop issue. The copolymers with three-dimensional network structure on the molecular chain can reduce the fluidity of the melt in the combustion process by chemical crosslinking. Copolyesters possess high melting viscosity, and can improve the flame retardancy and droplet resistance owing to the self-cross-linking reaction. This indicates that the cross-linked monomers may greatly deteriorate the crystal properties of the polyesters, especially the dosage of the cross-linked monomers, which affects the flame retardancy of the polyesters. Yi *et al.*^29–31^ introduced the concept of ionology into the field of phosphorus-containing copolymerisation. The flame-retardant phosphorus induces formation of a cross-linked polyester structure with ion aggregation, which greatly improves the polyester melt viscosity of the copolyester. The melt-spinning capacity of polyester is also significantly improved. Phosphorus-containing flame retardants and silicon-containing flame retardants have obvious synergistic flame retardancy and can reduce the generation of droplets, but the relevant synergistic flame retardant mechanism has not been studied in detail. Jouyandeh *et al.*^32–36^ targeted almost all properties of thermoset composites are more or less dependent on the situation of network formation in a system, proposes a dimensionless criterion which based on nonisothermal differential scanning calorimetry, known as ‘cure index’, for typical epoxy-based systems containing 0D nanoparticles, hereinafter referred to as “CI”. A comparative study was performed on curing potential and associated performance of low-filled epoxy/amine composites containing pristine halloysite nanotubes (P-HNTs), alkali-activated HNTs (A-HNTs), and silane-functionalized A-HNTs (F-HNTs), hydroxyl-rich halloysite nanotube (HNT)/silica nanosphere (SiO_2_) core/shell particles are functionalized with multi-arm hyperbranched polyethylenimine macromolecule and epoxy/Fe_3_O_4_ systems as model nanocomposite coatings, however, the synergistic cross-linking effect of additives on curing properties of the materials was not mentioned. Henri *et al.*^37–39^ used the flame retardancy index (FRI) method derived from conical calorimetric data of thermoplastic composites to investigate the flame retardancy performance. However, there is no mention of the effect of the synergistic cross-linking effect of flame retardants and inorganic compounds on flame retardant properties. Jouyandeh *et al.*^40^ synthesized pristine mica (Mica) and *N*-octadecyl-*N*′-octadecyl imidazolium iodide (IM) modified mica (Mica-IM) and characterized it used. The results indicated that the kinetics of the network formation and network degradation were correlated to demonstrate how molecular-level transformations can be viewed semi-experimentally, but the synergistic cross-linking of additives is not mentioned.

At present, flame retardants can improve the flame retardancy of polyester, but can not solve the problem of dripping. The polyesters are inherently flammable and become the core raw material of textile fabrics. In view of its flame retardant and anti-dripping properties are opposite, and flame retardancy and anti-dripping properties have remained key issues which should be resolved. To deal with these problems, this study proposes a combustion cross-linking method which improves the properties of the droplets by incorporating a synergistically cross-linkable structure of phosphorus-containing flame-retardant groups and acidic silica sol. The acidic silica sol will form a cross-linked network structure around the polyester during the combustion process, which can restrain the droplets and improve the anti-dripping properties. The groups containing phosphorous play a role in promoting the formation of carbon during the combustion process, and can have a synergistic effect with acidic silica sol, improve the flame retardant of polyester.

The flame retardant [(6-oxido-6*H*-dibenz[*c*,*e*][1,2]oxaphosphorin-6-yl)methyl]butanedioic acid, acidic silica, and polyester are prepared by using the copolymerisation method to obtain copolymers with flame retardant and anti-dripping properties. The flame-retardant properties and the behaviour of the copolymer are analysed by the LOI, UL-94, and cone calorimeter tests. Furthermore, the thermal degradation behaviour and the synergistically cross-linkable mechanism of copolymer are comprehensively studied.

## Experimental

### Materials

**Scheme 1:**
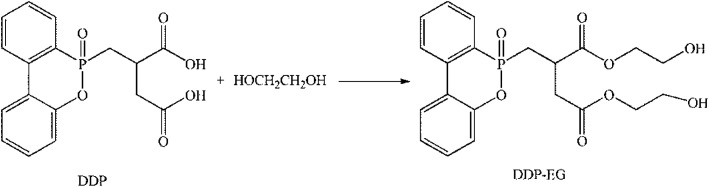
The modification schematic of flame retardant.

Purified terephthalate acid (PTA, Shenghong Petrochemical, Suzhou); ethylene glycol (EG, Liao Ning Chemical) and catalyst of ethylene glycol antimony (Sb_2_(OCH_2_CH_2_O)_3_, Liao Ning Chemical). [(6-Oxido-6*H*-dibenz[*c*,*e*][1,2]oxaphosphorin-6-yl)methyl]butanedioic acid (DDP, the structure of DDP see Scheme 1, Sino pharm Chemical Reagent, Beijing).

### Modification of flame retardant

The flame retardant of modification was prepared by esterification reaction using DDP and EG. In a typical experiment, 0.8 mol of DDP and 2.0 mol of EG were dissolved in 2000 mL flask, then the reaction temperature heated up to 180 °C under nitrogen protection for 6 hours. And then the system heated up to 195 °C, the residual ethylene glycol and water were distillated. At last, take out the product (DDP-EG) and put in samples of glass bottles. Schematic is shown in Scheme 1.

(Fig. S1† shows the ^1^H-NMR spectra of flame retardant DDP-EG and Table S1† shows the chemical shift of groups in the molecular structure. The thermal properties of DDP-EG were measured by TGA, Fig. S2† shows the thermal gravimetric curves of DDP and DDP-EG at nitrogen atmosphere. The analysis data is presented in Table S2†. These are provided with ESI†).

### Preparation of nano-SiO_2_ silica sol

Nano-SiO_2_ silica sol were prepared by the well-known StÖber's method as reported elsewhere. In brief, 100.0 mL ethylene glycol, 105.0 mL deionized water and 2.0 mL phosphoric acid were added to a 500 mL flask equipped with reflux condenser, phosphoric acid was used to control the pH of the reaction to 2.0. Then, 100.0 mL tetraethoxysilane was added into flask and stirred for about 30 min. Later, added 2.00 mL γ-(2,3-epoxypropoxy)propytrimethoxysilane. After feeding, the reaction as kept for 4 hours to get the nano-silica sol solutions. Then, the impurities such as water and ethanol were removed by the method of decompression distillation and obtained the nano-SiO_2_ silica sol with an average particle size of 180 nm.

### Preparation of copolyesters

**Scheme 2:**
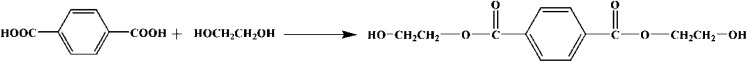
Esterification reaction.

**Scheme 3:**
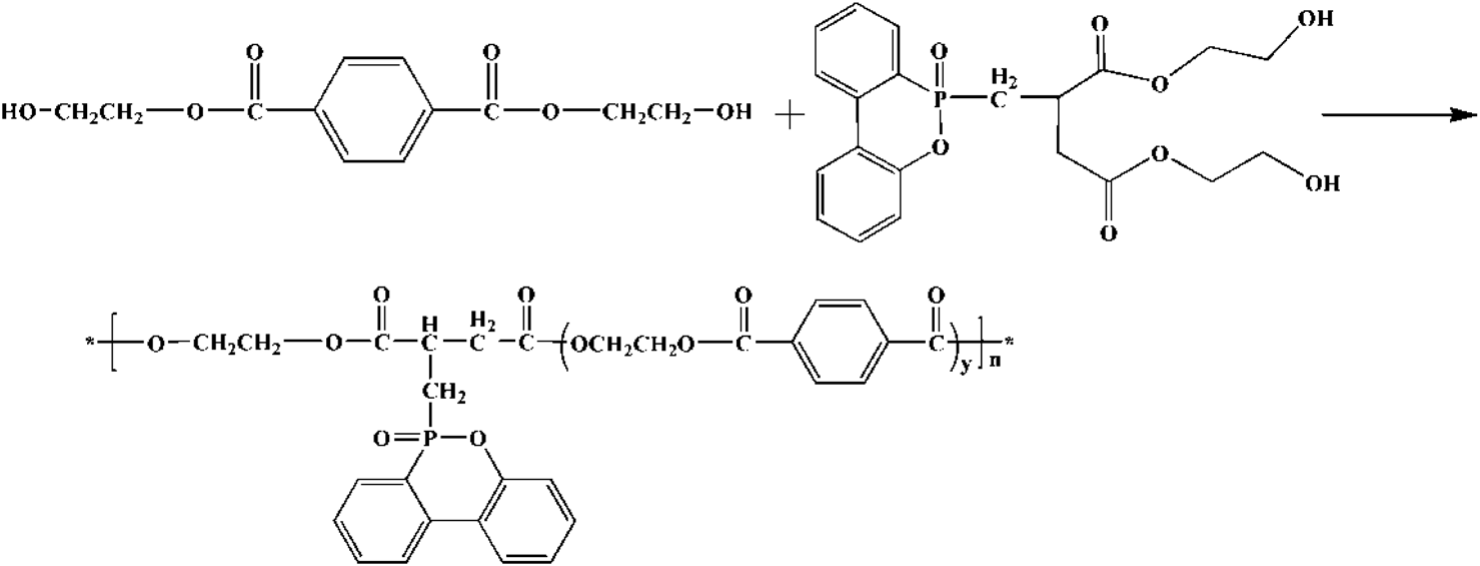
Polycondensation reaction.

Copolyesters was synthesized by macromolecular esterification were synthesized by macromolecular inter esterification method as shown in Scheme 2. The molar ratio of PTA to EG was used in the reaction. The temperature in the esterification process increased slowly from 230 °C to 245 °C, and the reaction pressure was 0.3–0.4 MPa, the reaction time was 3 hours. When the water yield reached the theoretical value, DDP-EG and SiO_2_ were added to the reactor, and ethylene glycol antimony was selected as the catalyst. Reaction temperature 250–260 °C, vacuum 300 Pa and maintain 60 min. Subsequently, polycondensation reaction temperature 270–275 °C, reaction time 2–2.5 h. Schematic is shown in Scheme 3. The modified silica sol–gel was prepared by distillation under 100 °C using nano-SiO_2_ silica sol and EG as raw material. The prepared copolyesters were numbered in the Table 1.

**Table 1:** The ratio of copolyester

Sample number	EG : PTA (mol/mol)	DDP-EG : PTA (mol/mol)	SiO_2_ (wt%)
Pure-PET	1.35 : 1	0	0
PETP	1.35 : 1	0.05 : 1	0
PETP-1Si	1.35 : 1	0.05 : 1	1.0
PETP-2Si	1.35 : 1	0.05 : 1	2.0
PETP-3Si	1.35 : 1	0.05 : 1	3.0

(The ^1^H-NMR spectra of modified PET polyesters (PETP is taken as an example) is shown in Fig. S3.† This is provided with ESI†).

### Pre-crystallization and drying of slices

Pre-crystallization of slices is carried out in a blast oven. First, pre-crystallization is carried out at 120–140 °C, keep for 3 h. Then a rotary blast oven is used for drying, keep for 24 h, and then naturally cool to room temperature to get the moisture content polyester chips below 50 ppm.

### Preparation of copolyester fiber

Preparation of copolyester fiber: the size of the silk board is 0.4 mm × 36, the spinning speed is 600 m min^−1^ and the spinning temperature range is 260–290 °C.

The preparation of the draft yarn: the copolyester fiber is drawn on a drafting machine, the temperature of the hot plate is 160 °C, the temperature of the hot plate is 80 °C, the draft ratio is 3.5 times, and finally the draft yarn is obtained.

### Characterization

The differential scanning calorimetry (DSC) test was performed on a TA Q20 DSC instrument. All samples were annealed (105 °C, 12 h) prior to the testing. In a nitrogen atmosphere of 50 mL min^−1^, heating rises from 30 °C to 300 °C (at a heating rate of 20 °C min^−1^). At 300 °C for 3 min, the thermal history can be eliminated. The temperature was then lowered to 30 °C (cooling rate was 10 °C min^−1^) and from 30 °C to 300 °C (heating rate was 10 °C min^−1^). Thermogravimetric analysis (TGA) measurements were carried out on a 209 F1 Iris thermal analyzer (TG, Netzsth, Germany) from 50 to 800 °C (heating rate of 10 °C min^−1^) in air atmosphere. Using INSTRON F563-44 according to ASTM D-638 at a speed of 5 mm min^−1^ with the mechanical properties of melt-spun fibers. The determination of the limit oxygen index was selected JF-3 the limit oxygen index chamber (Qionglai, China) method, using the standard ASTM D2863-0951. The American Association for Materials Testing A standard for vertical flame testing is the ASTM D1230-94 of the American Society of Materials Testing Adopt the instrument is CZF-5 type automatic vertical combustible cabinet (Nanjing Jionglei Analytical instrument Factory), the sample size is 125 × 13 × 3 mm^3^. A cone calorimeter was used to evaluate the combustion behavior of the sample. The selected standard was ISO 5660. The sample size is 100 × 100 × 3 mm^3^, the heat flux is 35 kW m^−2^ and the exhaust flux is 24 L s^−1^. The surface morphology of copolyester after combustion was characterized by scanning electron microscope (SEM) with JSM-5600LV (Japan), The acceleration voltage is 5 kV. EDX was tested on a quantax 400 (Bruker) system with a SEM attachment. All the samples were coated with a conductive layer of gold prior to SEM observation (the sample is sprayed with gold by spraying equipment to realize the coating of conductive layer). In order to explain the structure transition process of silica sol-side chain P containing copolyester in network acidic silica sol at high temperature, XPS was used to analyze the Si binding form of 2p orbitals of Si. FTIR spectra of carbon layer were obtained by means of a Nicolet 8700 FTIR spectrometer in the wave number range from 500 to 4000 cm^−1^ with the KBr pellet method. The Raman Spectra were measured at room temperature using spex Raman Instrument (1403, SPEX Co, USA) with excitation wavelength of 532 nm, the range of which is 600–3000 cm^−1^. The mechanical properties of the fibers were tested by XL-1 tensile tester. The length of the fibers was 250 mm, the tensile rate was 250 mm min^−1^, and the pretension was 5 cN. Each group of fibers was measured 20 times.

## Results and discussion

### Thermal characteristics of copolymers

**Fig. 1:**
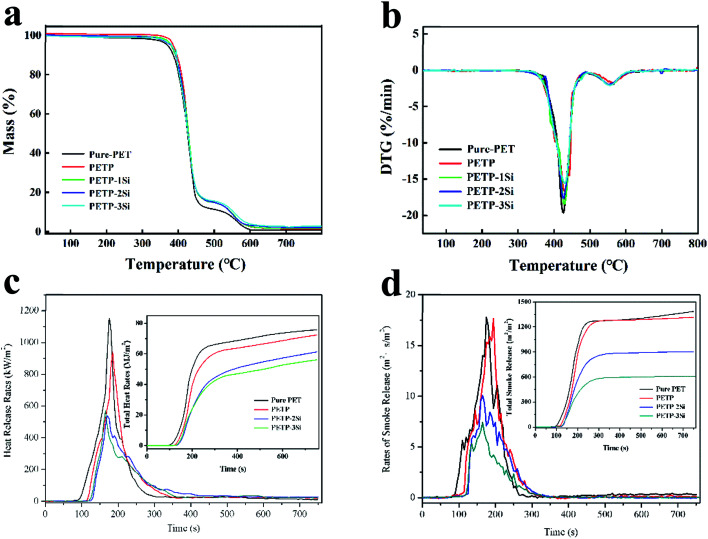
The copolyester of TGA (a) and DTG (b) on air atmospheres, HRR (c) and RSR (d).

The thermogravimetric analysis (TGA) is performed to evaluate the thermal stability of the samples to compare the effect of the SiO_2_ sol–gel and DDP on the thermal stability of the polyesters. Fig. 1a and b shows the results of the TG and the DTG curves under an air atmosphere. Table 2 presents detailed data such as *T*_5wt%_ is the temperature of 5 wt% weight loss, *T*_dmax_ is the temperature of maximum degradation, DTG_max_ is the maximum rate of decomposition.

**Table 2:** The TGA and DTG of copolyester (air atmospheres)

Samples	*T*_5%_ (°C)	*T*_dmax1_ (°C)	DTG_max1_ (% min^−1^)	*T*_dmax2_ (°C)	DTG_max2_ (% min^−1^)	Residual (wt%)
PET	370.3	425.8	−19.6	556.7	−2.0	0.81
PETP	386.5	428.9	−16.6	565.8	−1.6	1.51
PETP-1Si	380.0	425.8	−18.4	557.2	−1.9	1.70
PETP-2Si	380.9	428.3	−17.6	555.1	−2.0	2.27
PETP-3Si	381.2	429.9	−15.5	553.7	−2.1	2.72

It can be observed from Fig. 1a and b that all the samples exhibit a multi-step decomposition process. The degradation of the pure PET in air exhibits a two-step decomposition process, with an onset degradation of 370.3 °C, along with a maximum mass loss rate of approximately 425.8 °C due to depolymerisation by the β-H chain transfer reactions. The second degradation occurs at 556.7 °C due to the radical depolymerisation. The PETP initially presents a higher peak temperature (*T*_dmax1_) at the major weight loss stage and subsequently presents a higher peak temperature (*T*_dmax2_) at the minor weight loss stage when compared to the PET. The onset decomposition temperature increases significantly from 370.3 °C for the pure PET, to 386.5 °C for the PETP, which is consistent with the improved carbonisation in the presence of phosphorus. This indicates that the initial thermal stability of the PET is enhanced when a DDP-EG is introduced.

The PETP-Si samples present a different decomposition tendency when compared to the PETP, owing to the cross-linked nano-SiO_2_ and the water produced during the heating process. The *T*_5%_ and *T*_dmax1_ are gradually increased with the increase of the nano-SiO_2_ content, and the produced cross-linked nano-SiO_2_ inhibit the decomposition of the copolyester. The nano-SiO_2_ continues the cross-linking process at high temperatures, and the produced water accelerates the decomposition of the copolyester. It is observed that *T*_dmax2_ and DTG_max2_ gradually decrease with the increase of the nano-SiO_2_ content. Because under high temperature conditions, nano-silica will absorb heat to form a dense and uniform network silica structure, and phosphorus-containing flame retardants will form a carbon layer structure evenly distributed around the network silica structure to isolate oxygen and heat, this suggests that the nano-SiO_2_ can be used to inhibit the heat, delay the degradation and promote the carbon deposition.

### Flame retardancy behaviour of copolyesters

**Scheme 4:**
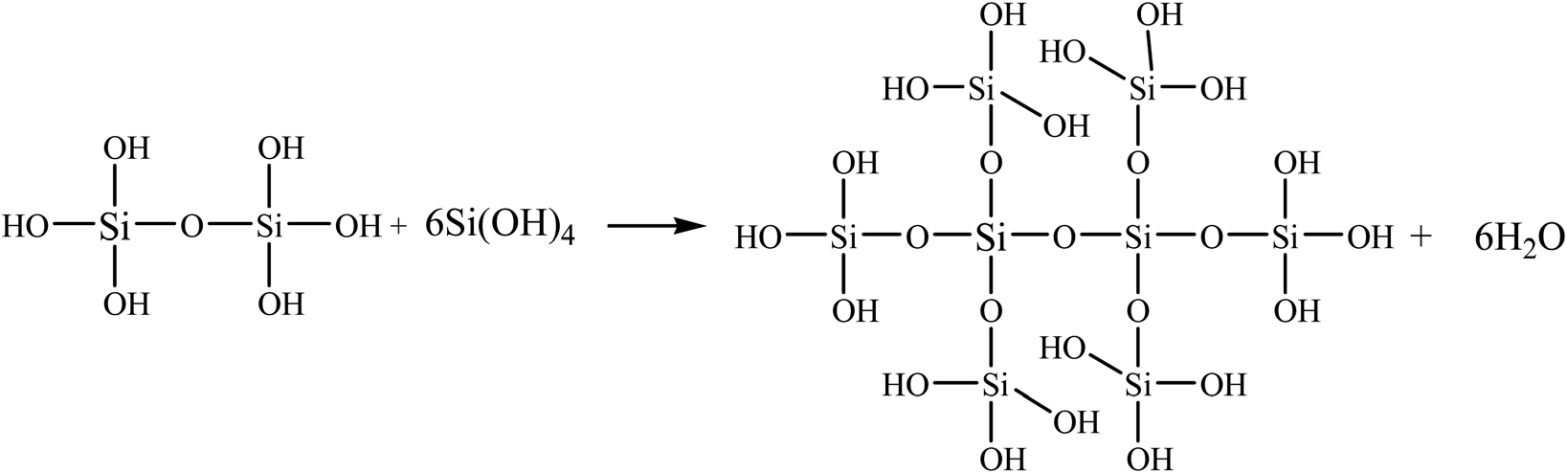
Cross-linked of SiO_2_ sol–gel particles.

The flammability characteristics of the modified copolyesters are evaluated by limiting the oxygen index and performing vertical flame tests. The results are summarised in Table 3. The pure PET readily ignites and burns with severe dripping, exhibiting high flammability. The LOI value of the PET is only 21.2% ± 0.2%, so the PET failed the UL-94 V test. The flame retardancy of the phosphorus-polyester with side chains is evidently improved with the incorporation of the SiO_2_ sol–gel particles. The LOI values increase from 32.5% ± 0.3% of the PETP to 34.8% ± 0.1% of the PETP-3Si. The UL-94 of the copolyesters is enhanced from V-2 of the PETP to V-0 of the PETP-3Si, and the second melt drop number (*d*_2_) is decreased from 9 to 2. This is because there are many hydroxyl structures on the surface of SiO_2_ sol–gel particles, the FT-IR spectra also shows that SiO_2_ sol–gel particles contain uncrosslinked hydroxyl groups, and silanol groups have the characteristics of high temperature crosslinking. The SiO_2_ sol–gel particles can be deeply cross-linked during the heating process, which reduces the fluidity of the copolyester melt and improves the anti-dripping property.^41–43^ Cross-linked of SiO_2_ sol–gel particles as shown in Scheme 4. Therefore, the SiO_2_ sol–gel particles with cross-linked structures enhance the flame-retardant properties and also effectively restrain the droplets.

**Table 3:** The flammability of modified PET polyester

Samples	Flammability		
	LOI (%)	Ignition cotton	UL-94
PET	21.2 ± 0.2	Y	—
PETP	32.5 ± 0.3	N	V-2
PETP-1Si	32.8 ± 0.2	N	V-0
PETP-2Si	33.8 ± 0.1	N	V-0
PETP-3Si	34.8 ± 0.1	N	V-0

The cone calorimeter test is a relatively comprehensive evaluation method which is used to induce the combustion processes under external thermal irradiation. A large amount of important real-time data recorded throughout the combustion process are heat, smoke, and carbonization. Fig. 1c and d shows the heat release rate (HRR), the total heat release (THR), the rate of smoke release (RSR) and the total of smoke release (TSR) curves of the copolyester, and the detailed data are listed in Table 4. The time to ignition (TTI) is used to determine the effect of the flame retardant on the ignitability. The DDP can effectively improve the high-temperature resistance of the copolyesters containing phosphorus when it is used as a high-temperature flame retardant, resulting in the increase of the ignition time (TTI) of the PETP from 81 s to 110 s. The high-temperature stability of the copolyester-containing phosphorus is increased with the introduction of silica sol, which improves its temperature resistance. The TTI of the PETP-2Si and the PETP-3Si is increased to 126 s and 121 s, respectively. At high temperature, the cross-linking of silica–hydroxyl groups absorbed heat, which made the network of acidic silica sol come to more stable shape structure in the process of transition. Therefore, the combustion process becomes slow, the combustion time increases greatly, and the combustion heat decreases greatly. The PK HRR and THR of PETP-2Si samples decreased to 539.8 kW m^−2^ and 61.1 MJ m^−2^, which were 46.8% and 80.9% of pure polyester samples, respectively. And the PK HRR and THR of PETP-3Si samples decreased to 510.0 kW m^−2^ and 59.2 MJ m^−2^, which were 44.2% and 78.4% of pure polyester samples, respectively.

**Table 4:** The cone calorimetric data of copolyester

Sample	Pure PET	PETP	PETP-2Si	PETP-3Si
Test start time (s)	69	70	70	72
Time to ignition (TTI) (s)	81	110	126	121
Time to flameout (s)	424	384	722	767
THR (MJ m^−2^)	75.5	72.2	61.1	59.2
PK HRR (kW m^−2^)	1153.6	940.3	539.8	510.0
PK SEA (m^2^ kg^−1^)	2670.7	2694.1	2240.4	1774.3
PK RSR (m^2^ s m^−2^)	17.8	17.7	10.1	7.4
TSR (m^2^ m^−2^)	1382.6	1305.1	834.7	623.7
FRI	—	1.7422	4.1078	4.3093
Color	—	Blue	Blue	Blue

The combustion of polyester has the characteristics of fast, violent and huge heat. A large amount of benzene and biphenyls and their derivatives, as well as other fuel gases, are produced due to incomplete combustion caused by free radical reactions during degradation. A large amount of smoke produced by the combustion of phosphorus-containing copolymers is attributed to the accelerated degradation of polyester by the combustion of phosphorus-containing copolymers. The maximum smoke rate (PK RSR) and total smoke content (TSR) of phosphate-containing copolymer were 940.3 m^2^ s m^−2^ and 17.7 m^2^ m^−2^, respectively. Acid silica sol and phosphonate have synergistic flame retardant effect. Phosphorus containing copolyesters decomposed by heat to accelerate cross-linking of reticular silica sol, forming a dense non-combustible carbon protective layer, and it promotes the isolation of heat and gas and has excellent inhibitory effect on the formation of combustible gas. The PK RSR and TSR of PETP-2Si samples were reduced to 10.1 m^2^ s m^−2^ and 834.7 m^2^ m^−2^, respectively, which were 56.7% and 60, 4% of the pure polyester samples. And the PK RSR and TSR of PETP-3Si samples decreased to 7.4 m^2^ s m^−2^ and 623.7 m^2^ m^−2^, which were 41.5% and 45.1% of pure polyester samples, respectively.

### Flame retardant index of copolyesters

The Flame Retardancy Index (FRI) is a dimensionless index, which takes into account PK HRR, THR, and TTI as the main cone calorimetry characteristics. The FRI was defined as the dimensionless ratio between the terms 
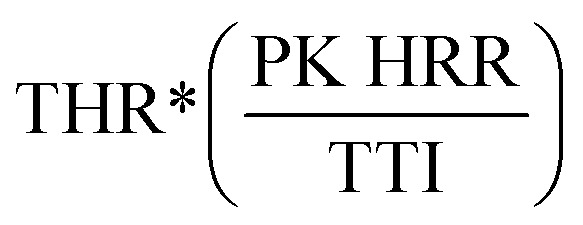
, with the nominator term corresponding to the neat polymer, and the denominator term to the polymer composite:^38^1
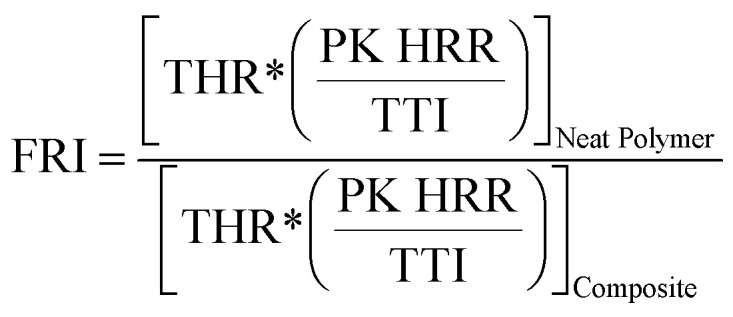


The flame retardancy of polymer composites can be qualitatively expressed using the FRI, with three possible cases, ‘Poor’ (FRI < 1, red), ‘Good’ (FRI < 10^1^, blue), and ‘Excellent’ (10^1^ < FRI < 10^2^, green), defined on a logarithmic scale (Table 4).

We can see in Table 4 that the FRI of the copolyesters containing phosphorous >1, indicating that the flame retardant has a better flame retardant effect in the flame retardant system; while the FRI of the copolyesters containing phosphorous–silicon is higher than that of the copolyesters containing phosphorous, indicating that under high temperature environment, the copolyesters containing phosphorous–silicon system is cured and crosslinked, which enhances the flame retardant properties of the copolyester.

### Morphology of residual char on the copolyester

**Fig. 2:**
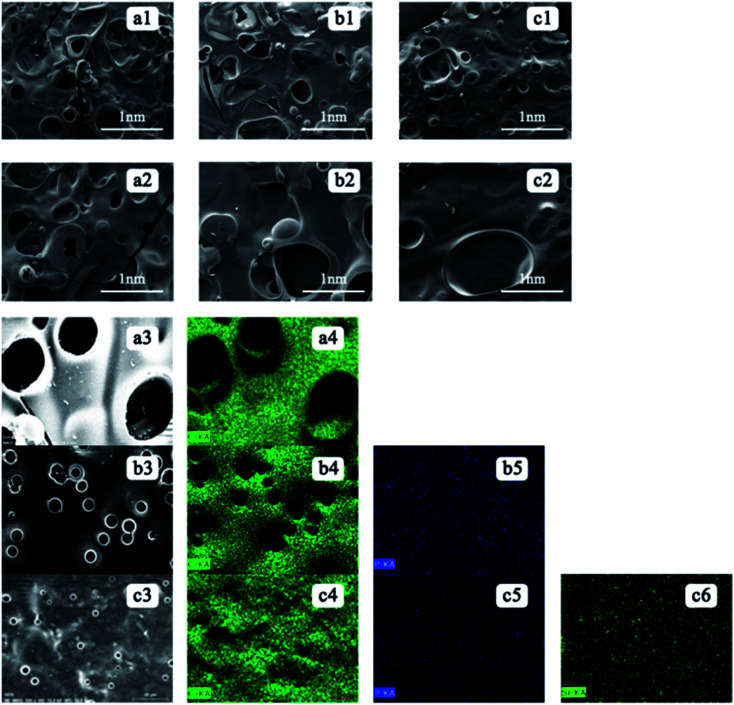
The SEM-EDX of residual char on copolyester ((a1), (a2), (a3) and (a4) was PET and, (b1), (b2), (b3), (b4) and (b5) was PETP, (c1), (c2), (c3), (c4), (c5) and (c6) was PETP-3Si).

The effect of flame retardant on carbon residue can be judged from the surface morphology of carbon residue after experiment. The char residue surfaces are analysed using SEM and EDX after performing the cone calorimetry. Fig. 2 shows the SEM images and the EDX analysis of the residual polyester for the samples of (a) pure polyester, (b) PETP, and (c) PETP-3Si. The distribution of the total copolyester residual char surface element, with a scanning time of 180 s, and distribution of elemental carbon, phosphorus and silica can be obtained from the EDX diagram. The poor compactness and thermal degradation of carbon residue surface produce combustible gas due to the violent combustion of pure PET and PETP. The pure PET carbon layer is loose, and irregular holes are observed on the surface of the carbon layer. The residue is observed to be a fractured char with multiple flaws and pores on its surface and is susceptible to cracking during burning. Large pores and smaller pore structures exist in the residual carbon. The number of holes in the carbon surface of the phosphorus-containing copolymer is reduced owing to the ability of the phosphorus-containing copolyester to accelerate the carbon formation. The residual char of the PETP is observed to be very compact and the pore size is also reduced. By comparison, the PETP-Si residual carbon is a very compact carbon slag, similar to the rough “carbonaceous clothing” covering the surface. The increase of silica nanoparticles is beneficial to the formation of dense and stable carbon layer, while inhibiting the expansion of carbon layer to form holes.

The carbon layer formed by pure PET is mainly carbon, while the carbon layer formed by PETP is carbon and phosphorus. With the addition of the acidic silica sol, the phosphorus and silicon elements are uniformly distributed in the carbon residue. The uniformity of the phosphorus element in the PETP-3Si sample is greater in the surface distribution and the signal of the phosphorus element becomes weaker, because the flame retardant DDP produces phosphoric acid, which accelerates the cross-linking of the reticular silica sol. Based on the XPS analysis, under the catalysis of acidic structure, the linear network (Si–O)_*n*_ structure rapidly transforms into the body SiO_2_ group between 400 °C and 500 °C, thermal crosslinking structure is formed.^24^ Cross-linked of SiO_2_ sol–gel particles as shown in Scheme 4. However, it is retained in the carbon residue which weakens the signal of the surface phosphorus element, indicating that the acid silica sol produces a synergistic effect with the flame retardant DDP. Therefore, the flame retardant promotes the formation of the cross-linking structure of silica sol, which can improve the compactness of the residual char through surface isolation and act as a condensed flame retardant.

**Fig. 3:**
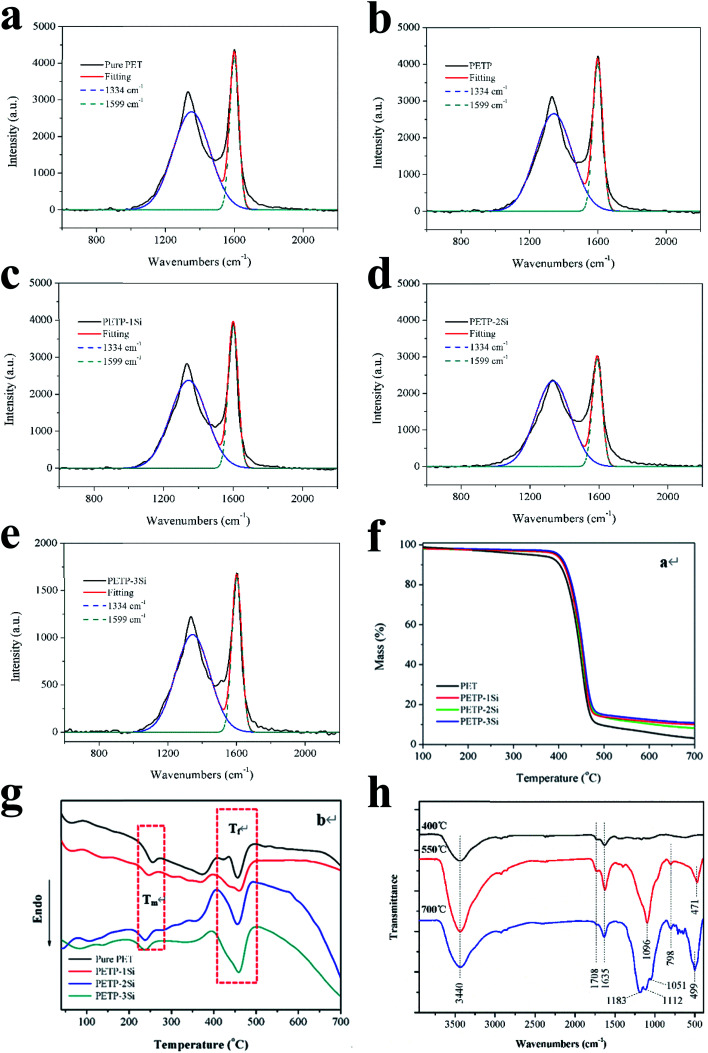
(a–e) The Raman of copolyester. The TG (f) and DSC (g) curve of copolyester. (h) The FTIR data of copolyester.

The combustion behaviour of the residual carbon of the copolyester containing the phosphorus–silica is analysed by Raman spectroscopy. Fig. 3a–e shows the Raman spectra of the residual carbon in the copolyesters. The D peak appears at 1334 cm^−1^, corresponding to the disordered carbon materials, and the G peak appears at 1599 cm^−1^, corresponding to an ordered carbon structure.^20,44^ The ratio of the areas of the G and the D peaks, *S*_G_/*S*_D_, is quantified to analyse the degree of the carbon layer order, with higher *S*_G_/*S*_D_ indicating a more ordered and denser carbon layer.^45,46^ The density of the carbonaceous structure increases with the addition of the acidic silica sol because the cross-linked silica sol promotes the graphitic carbon content. Furthermore, the half-peak width of the disordered graphite carbon layer in the residual carbon is reduced because the stability of the disordered graphite carbon layer structure increases. This indicates that the phosphorus containing copolyesters increase the content of the carbonaceous graphitic layer but decrease its stability. The silica sol structure also improves the structural content of the graphitic carbon layer and the stability of the disordered graphite carbon layer, indicating that the acidic silica sol has a synergistic effect on the phosphorus containing copolyesters. Therefore, the addition of acidic silica sol promotes the formation of dense carbon residue layer, and the results of SEM can be supported.

### Curing analysis

The curing reaction of the prepared copolyester was investigated by DSC technique. Fig. 3 shows the DSC thermograms of the copolyester at a heating rate of 20 °C min^−1^. The results on curing characteristics such as the total heat of cure (Δ*H*_∞_), the onset and *T*_g_ is the glass transition temperature endset cure temperature (*T*_onset_ and *T*_endset_), and the internal among which curing temperature changed (Δ*T* = *T*_endset_ − *T*_onset_) are given in Table 5. Qualitative cure analysis was performed by a so-called Cure Index (CI) criterion defined on the basis of nonisothermal DSC data as follows:^35^

2 CI = ΔH* × ΔT*

where3
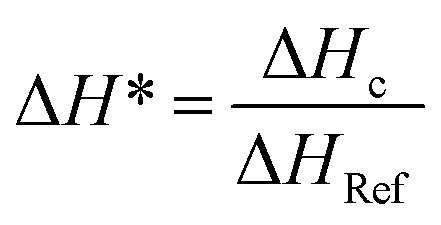
4
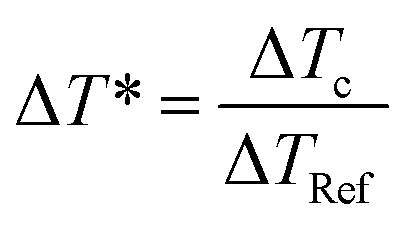


**Fig. 4:**
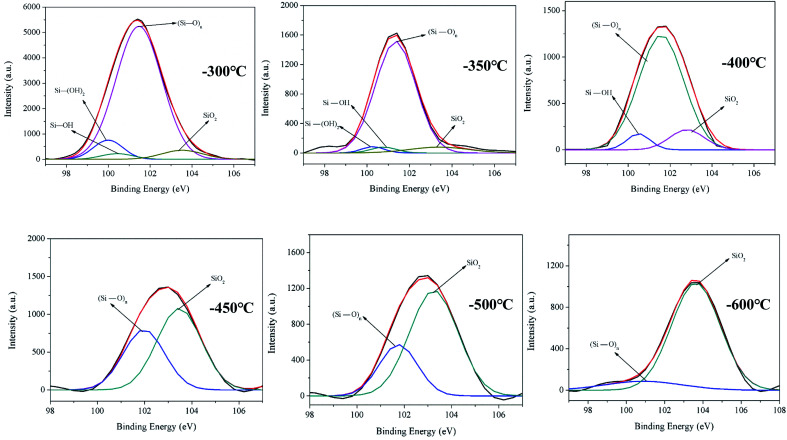
The XPS (Si 2p) of copolyester (PETP-3Si).

**Table 5:** Cure analysis of the prepared samples based on nonisothermal DSC data

Samples	*T*_onset_ (°C)	*T*_endset_ (°C)	Δ*T* (°C)	Δ*H*_∞_ (J g^−1^)	ΔT*	ΔH*	CI	Color
Pure-PET	141.9	197.3	55.4	43.9	n.a.	n.a.	n.a.	n.a.
PETP	82.1	160.2	79.0	26.4	1.4260	0.6014	0.8576	Red
PETP-1Si	91.3	164.9	73.6	28.9	0.9316	1.0947	1.0198	Green
PETP-2Si	101.5	169.3	67.8	32.6	0.8582	1.2348	1.0597	Green
PETP-3Si	112.0	174.0	62.0	34.1	0.7848	1.2917	1.0137	Green

In eqn (3) and (4), Δ*H*_c_ and Δ*H*_Ref_ are defined as the total heat release values of thermoset composites and the reference polymer, respectively. Likewise, Δ*T*_c_ and Δ*T*_Ref_ are the cure temperature intervals of thermoset composites and the reference polymer, respectively. According to this method, three possible cases were suggested for the CI, where CI > ΔH* (Good cure), ΔT* < CI < ΔH* (Excellent cure) and CI < ΔH* (Poor cure) could be calculated. The values of ΔT*, ΔH* and CI are calculated (Table 5), representing blue for Good cure, green for Excellent cure and red for Poor cure.

Compared with pure polyester, the total heat release of the copolyesters containing phosphorous decreases, while ΔT* > ΔH*, it indicates that the material has not been cured; by ΔT* < CI < ΔH*, shows that the curing degree of the copolyesters containing phosphorous–silicon is significantly improved compared with that of the copolyesters containing phosphorous, which proves that the curing behavior of the copolyesters containing phosphorous–silicon is improved after the addition of acidic silica sol, and all of them are Excellent cure, indicating a good curing degree (see Table 5).

### Thermal cross-linking mechanism of copolyester

The TG-DSC is used to observe the mass and the heat of the samples at high temperatures to analyse the mechanism of flame retardancy and anti-dripping for the copolyester. Fig. 3g and Table 6 show the TG-DSC curves of the samples. The addition of the acidic silica sol does not significantly affect the decomposition temperature of the copolyester in a nitrogen atmosphere, while the inorganic acidic SiO_2_ sol structure presents better high-temperature resistance. Therefore, the maximum decomposition temperature of the copolyesters containing phosphorous–silicon increases with the addition of the silica sol, increasing the carbon residue at 700 °C. The residual silica hydroxyl continues the cross-linking process to produce a body structure, and to avoid the initial chain-end hydroxyl ring-forming reaction of the copolyesters. The melting temperature (*T*_m_) and the decomposition temperature (*T*_f_) of the samples can be observed from the DSC curve of the copolyester. The copolyesters containing the phosphorus–silica undergo a hydroxyl-terminated cyclization reaction at the initial stage of polyester degradation upon the addition of the acidic silica sol; the first step of the endothermic process (*T*_f1_), is thus observed on the PET curve. The hydroxy-terminated cyclization at the initial stage of the polyester degradation is observed, and the *T*_f1_ of the pure PET is 423.7 °C. Additionally, the acidic SiO_2_ sols contain trace amounts of the cross-linking hydroxyl silicon structure, which combined with the acidic SiO_2_ sol, improves the polyester heat-resistant performance. The *T*_f1_ gradually increases with the increase in the silica sol, and the presence of the *T*_f1_ is not detected in the PETP-2Si samples, mainly due to the reticular structure of the silica sol, hindering the chain segment motion, thus hindering the hydroxyl-terminated cyclization process. Additionally, the hydroxyl terminating reacts with the silica sol at high temperatures to form a high-temperature resistant Si–O structure. Si–O bond can make SiO_2_ sol–gel particles have linear and bulk structure, and bulk silica hydroxyl structure mainly corresponds to network structure. According to XPS, under high temperature conditions, the total amount of hydroxyl decreases, the total amount of linear structure decreases, and the total amount of body structure increases. Cross-linked of SiO_2_ sol–gel particles as shown in Scheme 4. Therefore, the addition of acidic silica sol, increases the difficulty of the formation of the rings by the hydroxyl terminating, which improves the heat-resistant degradation performance of the copolyester. The second endothermic process (*T*_f2_) mainly involves the polyester chain segment degradation, the acidic silica sol cross-linking reaction, and the heat absorption with the addition of the acidic silica sol. The *T*_f2_ temperature change is minimal and the acidic silica sol add quantity is limited. This does not change the main chain of the polyester period of degradation of the endothermic reaction, but the heat absorption capacity increases with an increase in the acidic silica sol (Table 6). Thus, the acidic silica sol in the polyester thermal degradation process and the heat absorption, transform the ascending reticular structure to a more stable structure.

**Table 6:** The TG-DSC data of copolyester containing phosphorus–silica

Samples	*T*_5%_ (°C)	*T*_dmax_ (°C)	DTG (% min^−1^)	Residual (%)	*T*_m_ (°C)	*T*_f1_ (°C)	*T*_f2_ (°C)	Δ*H* (J g^−1^)
PET	422.1	445.4	−34.8	7.5	255.9	423.7	456.1	−172.0
PETP-1Si	424.4	455.2	−30.0	10.4	247.0	439.4	460.5	−310.3
PETP-2Si	419.9	450.1	−33.0	11.2	238.1	—	455.9	−383.2
PETP-3Si	422.9	455.3	−34.1	19.7	237.6	—	458.8	−494.5

To investigate the melting and crystallization behavior of these polyesters, DSC analysis for cooling and heating was carried out (curves shown in Fig. S4,† analysis results presented in Table S3†). These are provided with ESI.†

This study aims to better illustrate the synergistic cross-linking reaction of acidic silica sol and phosphorus-containing copolyester and to analyse the chemical structure of the carbon residue at different treatment temperatures. Fig. 3h shows the FTIR spectra of the carbon residue in the copolyester containing the phosphorus–silica. The absorption peaks at 1708 cm^−1^ and 1635 cm^−1^ correspond to the residual carbon, and can be always observed when the calcination temperature increases, indicating that calcination forms a residual carbon structure. The absorption peak at 3440 cm^−1^ corresponds to the –OH group, and the characteristic peak increases with the increase of the calcination temperature. The characteristic absorption peaks of PO_2_^−^, P–O, PO_4_^3−^, and P_2_O_7_^4−^ (approximately 1100 cm^−1^)^7,47^ and the bending vibration peaks of O=P–O (471 cm^−1^ and 499 cm^−1^) are also observed. These indicate that the phosphorus-containing copolyesters form phosphate and phosphite structures during the decomposition process; the strength of the acidic structure increases with the increase of the calcination temperature. The Si–O–Si antisymmetric stretching vibration peaks are observed at 1183 cm^−1^ and 1051 cm^−1^, and the symmetric stretching vibration peak is observed at 798 cm^−1^. The peak at 1183 cm^−1^ corresponds to a more stable silica structure, and the peak at 1051 cm^−1^ corresponds to the mesh linear silica structure.^48^ The linear Si–O–Si structure appears at 500 °C and 700 °C, and a more stable silicon dioxide structure is formed at 700 °C. Furthermore, the characteristic absorption peak is observed at 1112 cm^−1^ of the acid hydroxyl group and phosphate root is still present. Therefore, the copolyesters containing phosphorus–silica produce acidic phosphoric acid and phosphite through combustion. The phosphoric acid and the phosphite then catalyse the conversion of the acidic silica sol structure, thus generating the bulk and the linear silica structures.

The Si combination of Si 2p is used in the analysis to explain the structural transformation process of the reticulated acidic silica sol at high temperatures. The PETP-3Si samples are heated up to 300 °C, 350 °C, 400 °C, 450 °C, 500 °C and 600 °C at a rate of 20 °C min^−1^. The samples are kept at this temperature for 10 minutes, and the XPS test is then conducted. Fig. 4 shows the XPS spectra of the Si 2p orbital of the reticulated acidic silica sol after treatment. The binding energy of 100.4 eV is the Si–OH group, the binding energy of 101.0 eV is the Si–(OH)_2_ group, the binding energy of 101.4 eV is the linear reticular (Si–O)_*n*_, and the binding energy of 103.6 eV is the SiO_2_ group.^23,31^ As the treatment temperature increases, the Si–(OH)_2_ group and the Si–OH group of the cross-linking reaction in the sample are observed to decrease in turn. The Si–(OH)_2_ and the Si–OH groups disappear at the temperatures above 450 °C and the largest content of the linear network (Si–O)_*n*_ structure gradually decreases with the SiO_2_ transformation to the body structure between 400 °C and 450 °C. The SiO_2_ sharp transformation process of the linear network (Si–O)_*n*_ structure to the body is observed during the heat treatment. The reticular (Si–O)_*n*_ group of the copolyesters containing the phosphorus–silica rapidly transforms into the body SiO_2_ group between 400 °C and 500 °C. The transformation process of the structure also provides the droplet-proof properties of the copolymer.

(Table S4† shows the effect of acidic SiO_2_ sol on polymerization process and intrinsic viscosity. This is provided with ESI†).

### Mechanism for the synergistic flame retardant effect

**Fig. 5:**
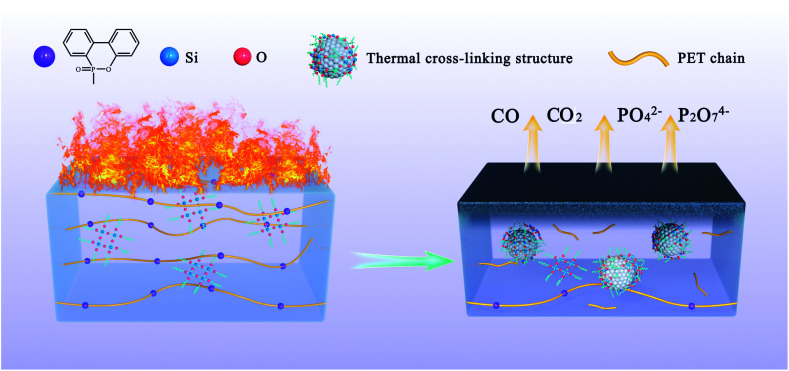
Scheme of proposed thermal cross-linking mechanism.

Based on the above analysis, a reliable mechanism of copolyesters containing phosphorus–silica thermal crosslinking was proposed. As shown in Fig. 5, the acidic silica sol is uniformly dispersed in the polyester system at the beginning. When burning, the acidic silica sol represented by the blue balls gradually changes from linear to a network structure, which is combined with the carbon residue formed by the groups containing phosphorus represented by the purple balls. A thermal cross-linking structure is formed, a dense carbon layer is formed on the surface of the polyester, and non-combustible gas is released to prevent the combustion from proceeding. Accurately, the phosphorous-containing copolyester chain are decomposed to a catalytic phosphoric acid and phosphite during combustion process of copolyesters containing phosphorus–silica. The phosphoric acid and phosphite structure promote the formation of dense carbon layer. It can isolate oxygen and prevent further combustion of copolyesters containing phosphorus–silica. Under the catalysis of acidic structure, the linear network (Si–O)_*n*_ structure rapidly transforms into the body SiO_2_ group between 400 °C and 500 °C. The anti-dipping of copolyesters containing phosphorus–silica is greatly improved due to the existence of thermal crosslinking structure.

### Spinnability of copolyester fiber

**Fig. 6:**
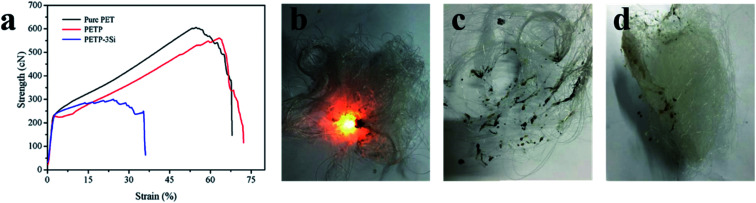
(a) The mechanical properties of copolymer fiber. (b–d) The burning photos of copolymer fiber ((b) was pure PET, (c) was PETP-3Si, (d) was PETP).

Fig. 6a shows the spinning and mechanical properties of copolyester fiber. The results show that the regularity of molecular segments is reduced by flame – retardant phosphorus-containing groups, which leads to the decrease of crystallinity. Therefore, the elongation at break increases and the breaking strength decreases. The reticular acid silica sol leads to the difficulty of copolyester spinning, and the tensile strength of PETP-3Si fiber decreased significantly, which was 1.5 cN per dtex. This problem can be solved by reducing SiO_2_ sol–gel particles size of silica sol, improving the dispersion and improving the polymerization degree of copolyester. At present, the copolyester fiber has low draw ratio and its strength can meet the application requirements of short fiber.

The LOI of the copolymer fiber were shown in Table 7. Pure polyester fiber has a LOI of 21.0 ± 0.3%, 31.9 ± 0.2% for PETP fiber, and 34.1 ± 0.1% for PETP-3Si. Copolyester fiber little change in LOI with copolyester chip. At the same time, in order to more intuitively study the flame retardant performance of copolymer fiber, continue the ignition of 12 s and remove the fire source digital photos, as shown in Fig. 6b–d. Pure PET fiber continues to burn with black smoke and pungent odor, while PTEP and PETP-3Si fiber leaves fire, but PETP fiber burns with black melting droplets. PTEP-3Si fiber only produces a small amount of melt in the ignition process, and there is basically no black smoke and pungent odor. So, PTEP-3Si fiber has excellent flame retardancy and anti-dripping.

**Table 7:** The mechanical properties and LOI of copolymers fiber

Samples	Fineness (dtex/36f)	Strength (cN per dtex)	Strain (%)	LOI (%)
Pure PET	197.3	3.0	55.1	21.0 ± 0.3
PETP	196.4	2.7	63.2	31.9 ± 0.2
PETP-3Si	195.2	1.5	25.1	34.1 ± 0.1

## Conclusion

Copolyesters with flame retardancy and anti-dripping were prepared by *in situ* polymerization of phosphorus-containing flame retardant (DDP) and acidic silica. Which resolved the problem that conventional methods of anti-droplet reduced the flame retardancy and may bring more smoke. Compared to pure PET, the smoke release rate of silicon–phosphorus co-polyester decreased by 45%, the heat release rate decreased by 78%, and the LOI was 34.8 ± 0.1%, UL-94 reached V-0 grade. The SEM and Raman results confirmed that the formation of dense carbon layer is due to the presence of acidic silica sol. At the same time, acidic silica sol can also inhibit the expansion of carbon residue by forming holes. Analyzed nonisothermally by DSC to investigate the qualitative and quantitative cure parameters of the systems. The Poor cure state was identified in the copolyesters containing phosphorus, by contrast, the Good cure state was under the condition of higher acidic silica sol, this also demonstrates the catalytic effect of cross-linking. TGA demonstrated that nanoparticle incorporation supports network formation, especially in the case of acidic silica sol. FRI-CI values indicate that the catalytic acid silica sol containing phosphorous flame retardant has good flame retardancy and curing degree from the microstructure. Furthermore, the synergistic cross-linking effect of copolyester was proved by TG-DSC. It was further confirmed by the XPS and FTIR results, copolyester containing phosphorus–silica produce acidic phosphoric acid and phosphite by combustion. Then, phosphoric acid and phosphite catalyze the conversion of acidic silica sol structure, thus generating bulk and linear silica structures. The copolyester fiber prepared by melt spinning has excellent flame retardant and anti-dripping. Which provides a simple and convenient method for preparing new effective flame retardant polyester is to use this combustion synergistic crosslinking effect to achieve flame retardant and anti-dripping modification of copolymers.

## Conflicts of interest

There are no conflicts to declare.

## Supplementary Material

RA-012-D1RA07410E-s001
