# Supplementary material for: Preparation and thermal cross-linking mechanism of co-polyester fiber with flame retardancy and anti-dripping by in situ polymerization
Source: RSC Adv. 2021 Dec 20;12(1):168–80. doi: 10.1039/d1ra07410e (PMC8978624; doi:10.1039/d1ra07410e)
Supplement: RA-012-D1RA07410E-s001 [file RA-012-D1RA07410E-s001.pdf]

Electronic Supplementary Material (ESI) for RSC Advances.
This journal is © The Royal Society of Chemistry 2021

Supporting Information

Preparation and Thermal Cross-linking Mechanism of Co-polyester Fiber with Flame Retardancy and Anti-dripping by in-situ Polymerization

Keyu Zhu [1], Zhenlin Jiang [1,2], Xiaotong Xu [1], Yun Zhang [1], Min Zhu [1], Jianghua Wang [3], Alex Ren [4]*

[1] *College of Chemistry and Chemical Engineering, Research Center for Advanced Mirco- and Nano-Fabrication Materials, Shanghai University of Engineering Sciences, Shanghai 201620, PR China*

[2] *Science and Technology on Advanced Ceramic Fibers and Composites Laboratory, National University of Defense Technology, Changsha 410073, PR China*

[3] *Jiangsu Guowanggaoke Fiber Co., Ltd, Suzhou 215228, PR China*

[4] *Shanghai Rongteng Packing Service Co. , Ltd. Shanghai 201620, PR China.*

* jiangzhenlin@sues.edu.cn, NO. 333 Longteng Rosd, Shanghai 201620, PR China.

1. Structure and properties of flame retardant

Fig.S1 shows the ^{1}H-NMR spectra of flame retardant DDP-EG and **Table.S1** shows the chemical shift of groups in the molecular structure. There is no proton signal for carboxylic group -COOH. Peak f (2.7ppm) is attributed to the proton signals of hydroxyl group -OH. Peak p (7.0-8.1 ppm) corresponds to the aromatic protons of the terephthalate units. The ratio of peak area between hydroxyl group and benzene is about 1:4. These results indicate that the esterification reaction is occurred between DDP and EG-terminated.

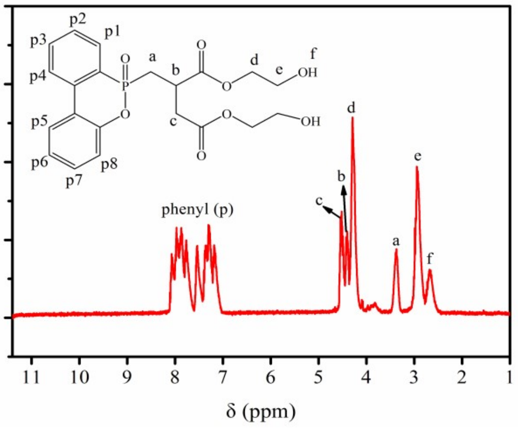

Fig. S1The ^{1}H-NMR of DDP-EG

Table. S1 The ^{1}H-NMR data of DDP-EG

Chemical shift	Integral area	Atomic ratio	Groups
7.0-8.1ppm	4.41	4	Phenyl (p)
4.5 ppm	1.08	2	c
4.4 ppm	0.64	1	b
4.3 ppm	2.43	4	d
3.4 ppm	1.07	2	a
2.9 ppm	2.35	4	e
2.7 ppm	1.00	2	f

The thermal properties of DDP-EG were measured by TGA. **Fig.S2** shows the thermal gravimetric curves of DDP and DDP-EG at nitrogen atmosphere. The analysis data is presented in **Table.S2**. $T_{5\%}$, T_{dmax} and DTG represent the initial decomposition temperature, the temperature at maximum weight loss rate and the maximum weight loss rate separately. It can be seen $T_{5\%}$, T_{dmax1}, and T_{dmax2} of DDP-EG are higher than that of flame retardant DDP, indicating DDP-EG with higher thermal propeties after esterification reaction. The reason why DDP-EG has good thermal performance is that DDP-EG has the EG-terminated structure. The excellent thermal properties of DDP-EG make it more beneficial to the copolymerization of PET.

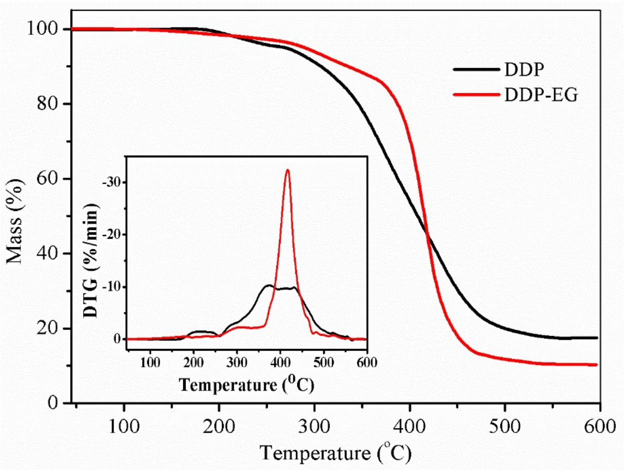

Fig. S2 The TGA and DTG of DDP and DDP-EG (at nitrogen atmosphere)

Table. S2 The TGA and DTG data of DDP and DDP-EG

Smaples	$T_{5\%}$ (°C)	$T_{dmax\ 1}$ (°C)	DTG $_{d1}$ (%/min)	$T_{dmax\ 2}$ (°C)	DTG $_{d2}$ (%/min)	$T_{dmax\ 3}$ (°C)	Residual (wt %)
DDP	269	225.1	-1.4	374.8	-10.3	431.3	17.5
DDP-EG	290	310.2	-1.7	417.2	-32.4	-	10.3

2. Structure and properties of flame retardant

The ^{1}H-NMR spectra of modified PET polyesters (PETP is taken as an example) is shown in **Fig. S3**. Peak a (8.20ppm) is attributed to the proton signals of aromatic protons of the terephthalate units. Peaks of multiple splitting i, h, g, f (7.1-7.5 ppm) corresponds to the proton signals of benzene ring on DDP-EG structure. Chmical shifts at 2.6 to 3.6 ppm correspond to the proton signals of methylene on ethylene glycol. The peaks of chmical shifts at 4.25 to 4.70 ppm attributed to the proton signals of diethylene glycol formed during polymerization process.The ^{1}H-NMR spectra of PETP suggests that the introcuction of flame retardant DDP-EG is incorporated into polyester chain segments by copolymerization. The prepared samples are the target products.

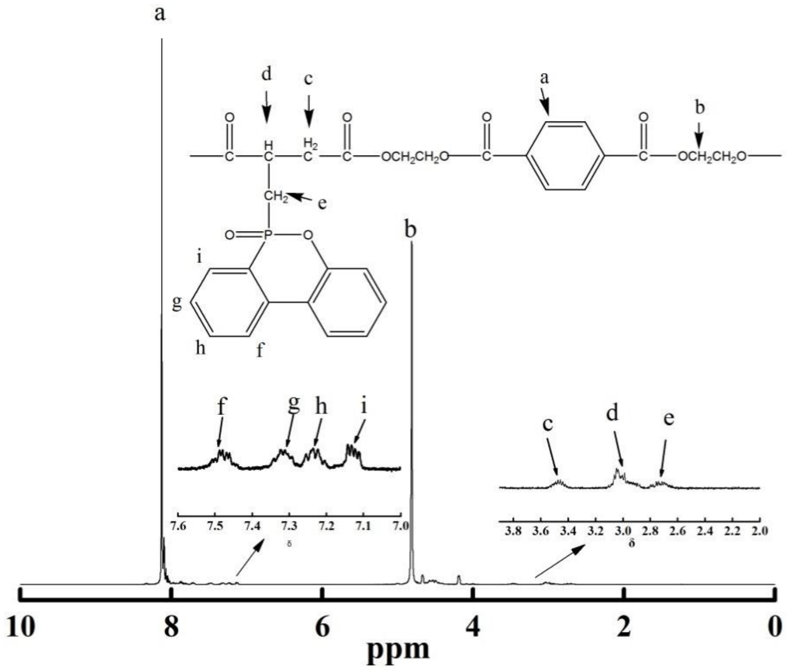

Fig. S3 The 1H-NMR spectra of PETP

3. Melting and crystallization behavior of copolyesters

To investigate the melting and crystallization behavior of these polyesters, DSC

analysis for cooling and heating was carried out (curves shown in **Fig. S4**, analysis results presented in **Table.S3**. The parameters of crystallization temperature, crystallization enthalpy, melting enthalpy and melting temperature are lower than those of pure PET due to the introduction of DDP with large steric hindrance into PET chain, leading the decrease of molecular chain regularity. With the increase of nano-SiO_2 silica sol content, the parameters of T_g of modified PET copolyesters show a decreasing trend caused by the nano-SiO_2 leading the free volume V_m between molecular chains increase and PET chain easier to move. The crystallization temperature T_c of modified PET copolyesters increases with the increase of the nano-SiO_2 content, indicating improvement of the nucleation rate. The faster the nucleation rate is, the better the crystallization property is. It is due to nano-SiO_2 silica sol as the funcation of crystallization nucleating agent in PETP matrix.

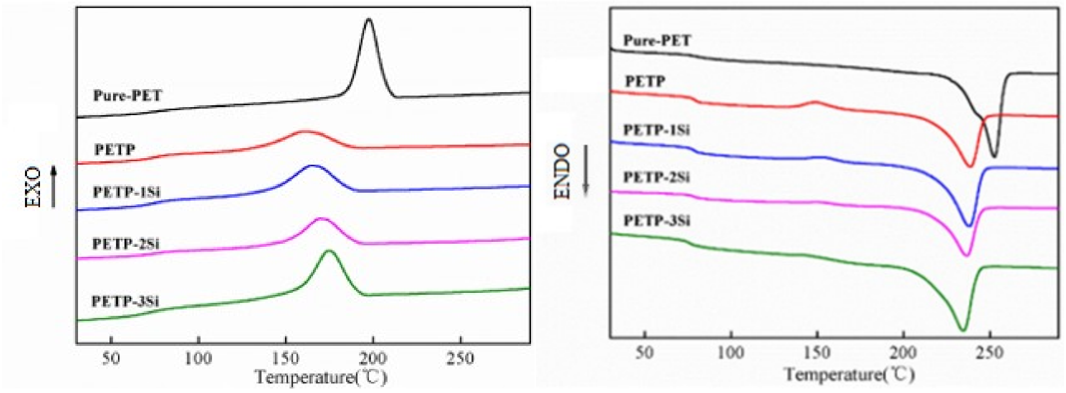

(a) crystallization behavior **(b) melt behavior**

Fig. S4 The DSC of copolyesters

Table. S3The DSC of copolyesters

Samples	T_g (°C)	T_m (°C)	ΔH_m (J/g)	T_c (°C)	ΔH_c (J/g)	ΔT_c (°C)	D (°C)
Pure-PET	82.1	252.7	41.8	197.3	43.9	55.4	10.7
PETP	78.7	239.2	32.2	160.2	26.4	79.0	26.8
PETP-1Si	77.8	238.5	33.6	164.9	28.9	73.6	21.9
PETP-2Si	76.3	237.2	34.7	169.3	32.6	67.8	20.2
PETP-3Si	75.2	235.1	32.5	174.0	34.1	62.0	16.4

4. Characteristic viscosity of copolymers

Table.S4 shows the effect of acidic SiO_2 sol on polymerization process and intrinsic viscosity. The acidic SiO_2 sol does not catalyze the degradation of polyester, but the network structure of acid SiO_2 sol can improve that the entanglement effect of macromolecular chain and increase the characteristic viscosity of silicon - phosphorus copolymer ester. However, the structure of entangled macromolecules affects the transesterification reaction, which leads to the decrease of molecular weight and intrinsic viscosity. Using inert inorganic acid silica sol as anti-fuse modifier can not only guarantee good molecular weight, but also guarantee the flame retardancy of silicon-phosphorus co-polyester. Using the cross-linking characteristics of acidic reticulated silica sol, the network structure of silica sol was transformed into cross-linked structure, and the anti-melting modification of silicon-phosphorus copolyester was achieved.

Table. S4 The intrinsic viscosity of copolyester

Samples	DDP (mol%)	Nano-SiO_2 (wt%)	Intrinsic viscosity ($dL\cdot g^{-1}$)
Pure PET	0	0	0.660
PETP	5	0	0.728
PETP-1Si[d]	5	1	0.631
PETP-2Si	5	2	0.530
PETP-3Si	5	3	0.525
